# Draft genomes of three bacteria capable of growing on cocamidopropyl betaine or *N*,*N*,*N*-trimethylglycine (betaine): *Cupriavidus* sp. CuC1, *Variovorax* sp. VaC1, and *Pseudomonas* sp. PsB

**DOI:** 10.1128/mra.01269-24

**Published:** 2025-03-10

**Authors:** Jinha Kim, Arum Han, Kung-Hui Chu

**Affiliations:** 1 Zachry Department of Civil and Environmental Engineering, Texas A&M University, College Station, Texas, USA; 2 Department of Electrical and Computer Engineering, Texas A&M University, College Station, Texas, USA; The University of Arizona, Tucson, Arizona, USA

**Keywords:** cocamidopropyl betaine, betaine, biodegradation

## Abstract

We report three draft genome sequences of *Pseudomonas* sp. PsB, *Cupriavidus* sp. CuC1, and *Variovorax* sp. VaC1. *Pseudomonas* sp. PsB can grow on *N*,*N*,*N*-trimethylglycine (betaine), but not on cocamidopropyl betaine (CPB), as a sole carbon source. *Cupriavidus* sp. CuC1 and *Variovorax* sp. VaC1 can grow only on CPB.

## ANNOUNCEMENT

Cocamidopropyl betaine (CPB) is a surfactant used in cosmetic products (1), anaerobic digestion processes (2), nanoparticle stabilization (3), and aqueous film-forming foam (AFFF) formulants (4, 5). *N*,*N*,*N*-trimethylglycine (betaine) could potentially be used as an anti-freeze agent in AFFF concentrates (4), is a common osmolyte, and known as a crucial metabolite in bacteria (6, 7). Here, we report three draft genomes of *Pseudomonas* sp. strain PsB, *Cupriavidus* sp. strain CuC1, and *Variovorax* sp. strain VaC1 isolated from CPB- or betaine-enriched soil cultures capable of degrading fluorotelomer-based precursors (8).

The soil enrichment culture was first established in a 20 mL vial containing 2 mL ammonia mineral salt (AMS) media (9), 0.05% CPB as the sole carbon source, and homogenized sandy soil (0.1 g) collected at a depth of 91–203 cm near a former Air Force base (10). The vial was shaken at 160 rpm at room temperature for a week before daily subcultures using 5% passage of the enriched culture to a new vial. The CPB enrichment was used as inoculum to establish a betaine-degrading culture with 5 mM betaine in AMS media. Strains CuC1 and VaC1 were isolated from AMS agar plates amended with CPB. Strain PsB was isolated with betaine as the carbon source. The isolates grew in AMS media with 0.05% CPB (CuC1 and VaC1) or 5 mM betaine (PsB). The FastDNA SPIN Kit for Soil (MP Biomedicals, CA) was used for extracting genomic DNAs (gDNAs). The 16S rRNA genes were amplified using the 24F/1492R primer set. Eton Biosciences (San Diego, CA) performed the paired-end Sanger sequencing. The trimmed paired-end reads were merged through VSEARCH (11). Through BLAST (12), CuC1 (PQ396171), VaC1 (PQ443692), and PsB (PQ443691) were closely associated with *Cupriavidus* sp. BD0822-70LYW1-18 (LC515384.1; 100%), *Variovorax* sp. AFS004558 (OP986438.1; 99.57%) and *Pseudomonas plecoglossicida* NBRC 103162 (NR_114226.1; 99.93%), respectively.

Cell suspensions originally inoculated with the same colonies were again used for gDNA extraction. The gDNAs were extracted using the PureLink Genomic DNA Kit (Life Technologies, CA) and quantified using Qubit Fluorometer 4.0 (Invitrogen, CA). The gDNA quality was checked with the D1000 ScreenTape Assay Kit (Agilent, CA) on the 4200 TapeStation (Agilent, CA). Libraries were prepared and quality-controlled with the NEBNext Ultra II DNA Library Preparation Kit (New England Biolabs, MA) and the KAPA Library Quantification Kit (Roche, Basel, Switzerland), respectively. Paired-end 2 × 150 bp sequencing was performed through the Illumina NovaSeq X platform (Illumina, CA). Raw reads were trimmed via Trimmomatic v0.4 (13). The genomes were *de novo* assembled using SPAdes v4.0.0 (14). Contigs with length < 500 bp and 77-mer coverage < 5 were removed (https://github.com/ECBSU/oneliners). Statistical information was retrieved using QUAST v5.2.0 (15). Completeness and contamination were checked through CheckM2 v1.0.2 (16). The genome coverages (i.e., total length of trimmed and aligned pair-end reads/total assembly length) were calculated using SAMtools v1.21 (17) after the paired-end reads were aligned against the filtered contigs using Bowtie2 v2.5.3 (18). Contigs were submitted to NCBI and annotated through the NCBI Prokaryotic Genome Annotation Pipeline v6.8 (19). The genomic features of the isolated strains are provided in Table 1.

**TABLE 1:** Genomic features of strains CuC1, VaC1, and PsB

Features	CuC1	VaC1	PsB
Sequence Read Archive accession #	SRR31344975	SRR31348096	SRR31350891
Total sequenced library length (bp)	5,055,303,900	4,473,307,200	4,801,127,400
Assembly accession #	GCA_044627735.1	GCA_043658315.1	GCA_043658375.1
Number of contigs	473	33	106
Assembly length (bp)	10,946,258	6,938,278	6,425,053
Number of pair-end reads (trimmed and aligned)	14,096,932	12,810,382	14,066,831
Genome coverage	381×	545×	650×
Contig *N_50_* (bp)	57,476	987,950	168,335
Contig *L_50_* (bp)	64	3	1
Guanine–cytosine content (%)	64.5	67	62.5
Completeness (%)	100	100	100
Contamination (%)	6.42	0.63	0.3
Number of coding sequences (CDS)	10,382	6,390	5,953
Number of CDS with proteins	9,913	6,358	5,838
Number of complete rRNA genes (5S, 16S, 23S)	3, 1, 0	1, 0, 1	2, 0, 0
Number of predicted tRNAs	63	67	44

## Data Availability

Whole Genome Shotgun projects have been deposited in GenBank under accession numbers JBIUH000000000.1 (CuC1), JBIGLT000000000.1 (VaC1), and JBIGLU000000000.1 (PsB). BioProject accession numbers are PRJNA1172179 (CuC1), PRJNA1172180 (VaC1), and PRJNA1172183 (PsB). BioSample accession numbers are SAMN44265317 (CuC1), SAMN44265322 (VaC1), and SAMN44265324 (PsB). Sequence Read Archive (SRA) accession numbers are SRR31344975 (CuC1), SRR31348096 (VaC1), and SRR31350891 (PsB).
